# Development of an ammonia-biodiesel dual fuel combustion engine's injection strategy map using response surface optimization and artificial neural network prediction

**DOI:** 10.1038/s41598-023-51023-1

**Published:** 2024-01-04

**Authors:** R. Elumalai, K. Ravi, P. V. Elumalai, M. Sreenivasa Reddy, E. Prakash, Prabhakar Sekar

**Affiliations:** 1 School of Mechanical Engineering, VIT University, Vellore, India; 2 Department of Mechanical Engineering, Aditya Engineering College, Suramplaem, 533437 India; 3 Department of Mechatronics Engineering, Rajalakshmi Engineering College, Chennai, 602105 India; 4 Department of Mechanical Engineering, Wollo University, Dessie, Ethiopia

**Keywords:** Mechanical engineering, Environmental sciences

## Abstract

The study intends to calibrate the compression ignition (CI) engine split injection parameters as efficiently. The goal of the study is to find the best split injection parameters for a dual-fuel engine that runs on 40% ammonia and 60% biodiesel at 80% load and a constant speed of 1500 rpm with the CRDi system. To optimize and forecast split injection settings, the RSM and an ANN model are created. Based on the experimental findings, the RSM optimization research recommends a per-injection timing of 54 °CA bTDC, a main injection angle of 19 °CA bTDC, and a pilot mass of 42%. As a result, in comparison to the unoptimized map, the split injection optimized calibration map increases BTE by 12.33% and decreases BSEC by 6.60%, and the optimized map reduces HC, CO, smoke, and EGT emissions by 15.68%, 21.40%, 18.82, and 17.24%, while increasing NOx emissions by 15.62%. RSM optimization with the most desirable level was selected for map development, and three trials were carried out to predict the calibrated map using ANN. According to the findings, the ANN predicted all responses with R > 0.99, demonstrating the real-time reproducibility of engine variables in contrast to the RSM responses. The experimental validation of the predicted data has an error range of 1.03–2.86%, which is acceptable.

## Introduction

The world energy organization is hastening the transition to zero carbon emissions and promoting innovative energy sources. Consumption of fossil fuels is declining, while the need for green, renewable energy is increasing, altering the foundation of energy requirements^1^. Global energy is transitioning to carbon-free and low-carbon energy, which involves reorganizing the foundations of the fuel sector and decarbonizing the global power sector. As the energy sector quickly decarbonizes, internal combustion (IC) engines encounter a dilemma that has never been seen before. The best solution to address the IC engine decarbonization problem is to identify alternative, sustainable carbon-free and low-carbon energy sources^2^. Various sustainable energy sources, such as alcohol, ether, and ester fuels, have been extensively researched in recent years. These energy sources may greatly decrease the use of fossil energy sources, but they are merely low-carbon energy sources. Despite being less carbon intensive than fossil energy sources, engine exhaust still produces large carbon emissions after use. One of the most significant methods to accomplish zero-carbon emissions from IC engines is to use ammonia and hydrogen as fuel, a kind of zero-carbon fuel, instead of fossil fuel^3^. Backfire and knocking limited the use of hydrogen energy in IC engines. On the other hand, achieving pure ammonia compression ignition is difficult due to the poor ignitability, flame speed, and combustion characteristics and the higher ignition temperature^4^. As a result, the majority of contemporary research on ammonia energy is conducted using diesel^5^, biodiesel as supplementary fuel for ignition^6^. The hydrogen density of ammonia is greater, and its cost of manufacture is very cheap^7^. An experiment examined the impact of injection time on a dual-fuel ammonia CI engine. The result demonstrates that advancing injection timing increases NOx emissions while decreasing NH_3_ exhaust emissions^8^.

By increasing the intake temperature, Niels Forby et al.^9^ increased premixed ammonia energy up to 98.5%, and the rest is direct-injected n-heptane. When the intake temperature is raised, the ammonia energy fraction also increases, and NH_3_ exhaust emissions decrease, which may shorten the ignition delay.

Sechul^10^ studied a dual-fuel engine that used ammonia fuel to replace CNG. The result reveals that 50% of ammonia substitution reduced CO_2_ emissions by 28%. Yousef et al.^11^ examined the effect of split injection in an ammonia-diesel powered dual-fuel engine. In comparison to neat diesel, raising the ammonia proportion dropped BTE and engine exhaust NOx emissions, but advanced diesel injection reduced CO_2_ emissions^12^. Furthermore, split injection of pilot fuel might lower NH_3_ exhaust emissions while increasing BTE.

Due to the diesel engine's high efficiency and longevity, it has been widely used in industrial applications, including power plants and many kinds of transportation^13^. Furthermore, growing worries about petroleum depletion and stringent emissions rules compel the creation of more cost-effective and environmentally friendly diesel engines^14^. Outside of an after-treatment system, the soot and nitrogen oxide emissions generated by diesel engines are particularly difficult to detect all at once. These regulations and concerns push engine innovation in the direction of increasing engine efficiency while lowering polluting emissions. Furthermore, as better solar fuels and biofuels become accessible, technical developments should improve tolerance for alternate fuels in power generation and transport systems. LTC (Low-Temperature Combustion) technologies are being developed in order to reach the goal of a fuel-independent, efficient, white engine^15^. Inside the cylinder, the temperature is maintained lower than in CDC (conventional diesel combustion)^16^. As a consequence, the rate of NOx generation decreases, and heat transfer losses reduce. Furthermore, these methods mix air and fuel effectively, avoiding fuel rich zones. As a consequence, soot and nitrogen oxides may be reduced without affecting efficiency. RCCI investigates one of many LTC techniques used in CI engines to maximize efficiency while minimizing the NOx/soot trade-off. It is commonly used in conjunction with two types of reactive fuels. LRF is introduced through the intake manifold during suction, whereas HRF is directly injected into the cylinder at the end of compression^17^.

Curran et al.^18^ examined the RCCI effect on a dual fuel CI engine and understood that BTE rose 7%, NOx emissions decreased, and carbon emissions increased relative to pure diesel conventional mode. Gharehghani et al.^19^ examined the impact of CH_4_/biodiesel and CH_4_/diesel-powered RCCI. The result revealed that biodiesel's in-built fuel oxygen enhanced BTE while NOx emissions dropped. Paykani et al.^20^ examined the effect of split injection techniques of a CH_4_/diesel dual fuel RCCI engine. It was discovered that the pre-injection and pilot mass are critical elements influencing the performance of an RCCI combustion. Liu et al.^21^ examined the RCCI impact using natural gas and diesel powered RCCI using a split injection approach. The results demonstrate that advancing main injection reduces CO and HC emissions while increasing knock propensity and NOx emissions. The split injection method causes RCCI mode combustion to be exothermic, resulting in a double peak heat release. When pre-injection is advanced, the peak cylinder combustion pressure improves, and CO and HC emissions decrease by 56% and 43%, respectively. Kumar Subramani et al.^22^ examined the effect of petrol and diesel powered RCCI. The findings showed that increasing the LRF energy input from 10 to 60% increased cylinder pressure and thermal efficiency while reducing CO_2_ emissions when related to the standard diesel combustion. As a result, it is necessary to transition from conventional nonrenewable fuels to carbon–neutral renewable fuels in order to fulfill emission standards. In this context, biofuels such as biodiesel are viable power options. Table 1 shows previous research on dual-fuel engines.

**Table 1:** Results of the dual fuel engine study reported in the literature.

References	Port fuel	Direct injection	Engine particular	Combustion indices		Performance indices		Emission indices	
				Increased	Decreased	Increased	Decreased	Increased	Decreased
^23^	Hydrogen and CNG	Pongamia pinnata biodiesel	1-C, 4-S, DI, 1500 rpm, 5 kW	CPmax, HRRmax	ID, CD	BTE-34.6%	BSFC	NOx-2.56 g/kWh	HC-0.54 g/kWh CO-5.25 g/kWh
^24^	Cotton seed biodiesel	Biodiesel blends	1-C, 4-S, DI, 2300 rpm, 5.5 kW	–	–	BTE-36%	BSFC-27%	HC, CO2-17%	NOx-24%
^6^	Ammonia 40%, 50%, 60% and 70%	Diesel	4-S, 1-C, 1500 rpm, 5.5 kW	CPmax, HRRmax		ITE		CO, HC, NOx	NH3-13%
^25^	Natural gas	Diesel	1-C, 4-S, DI, CRDI, CR 11.5 to 19	CPmax, HRRmax		BTE	BSEC	CO, HC	NOx
^26^	Cotton seed oil biodiesel	n-Pentanol	1-C, 4-S, DI, 1500 rpm, 5.4 kW	–	CPmax-5%, HRRmax-3.7%	BTE-2.41%	BSFC	HC-4.5%, CO-11%	NOx-43%

### Calibration map for IC engine

The use of machine learning in engine optimization and calibration is becoming increasingly widespread and successful^27^. It is possible to use machine learning models to engine performance prediction and calibration. For virtual calibration, an accurate, quick, and simple prediction model can be utilized^28^. The basic idea behind (ANN) is to recreate the computational capabilities of the brain in order to simulate the operation of a system^29^. ANN promises the ability to handle highly complex linear and non-linear systems^30^. The design of experiments (DoE) is a systematic approach for establishing a link between a process inputs and outputs, and it is effective for estimating the effects of independent variables on the responses^31^. The map-based injection control technique is the universal technique due to its simplicity. The ECU's lookup table regulates present engine control settings depending on the required injection parameters. The old method of CRDi lookup tables requires a large number of trial-and-error tests, which is difficult for contemporary engines. ANN-based validation is acknowledged as a cutting-edge method for enhancing calibration effectiveness and accuracy with minimal trial via Design of Experiment (DoE). The predictability of the ideally calibrated points is strongly dependent on the model's prediction performance in the modal based calibration approach. The selection of an appropriate DoE contains all of the necessary information to develop accurate parametric and non-parametric engine empirical behavior models. The development of engine models for different biofuels has been the subject of several research in recent years. For fitting linear, complete, or partial quadratic polynomial models, the Response Surface Methodology is typically employed in conjunction with DoE and least squares regression analysis. Also, several new neural network modeling techniques showed significantly better prediction than RSM. The majority of these ANN models primarily concentrated on steady state functioning, although the DoE almost guarantees its estimate in conformity with transitory emissions. Finding the most effective injector control factors for biofuel is the focus of many articles. They are only discovered at a certain engine operating point, which is inadequate for practical use.

It heavily emphasizes the creation of global ideal calibration procedures and comprehensive engine mapping. The two-stage model approach is used to improve the engine's multifunctional working, where integrating all optimal points will be difficult. As engine variables rise, an increase in data makes it possible to continuous calibration management.

The global model approach reduces calibration time significantly compared to the local model technique. It is proposed in this study to use new fuels that are dependable for diesel engines. Similarly, optimization and calibration approach are provided to fill an ideal map with assured objective constraints, resulting in lower emissions and higher fuel efficiency. The details of previous studies on various facets of engine characteristic calibration map construction for different biofuels are shown in Table 2.

**Table 2:** Literature on calibration procedures used for optimising the diesel engine.

References	Objective	Fuel type	Model type	Calibration approach	Results
^32^	Engine design and operating parameters	Producer gas	RSM	Desirability function approach	The optimal conditions are determined to be CR 16 and 100% load The corresponding performance indices were BTE↑21.02%, BSEC↓34.16 MJ/kWh, CO↓0.092% volume, HC↓17.4 ppm, CO_2_↑2.1% volume, and NOx↑4.5 ppm The mean values of R^2^ were 95%–99% and the determination coefficient was 0.72
^33^	Split injection parameters	Diesel and orange peel oil (OPO) blends	RSM and DNN	Multi-objective optimization using the desirability function approach	Optimal condition is determined as 50% load for OPO20. The corresponding performance indices were BTE↑14.06%, smoke↓85.67%, HC↓83.15%, CO ↓ 11.3%, and NOx↑18.71% with R^2^ values above 0.9417
^34^	Split injection parameters	Diesel	Genetic algorithm	Multi-objective optimization	BSFC, NOx, and CO with R^2^ values of 0.981, 0.979, and 0.968, respectively. After optimization, the BSFC↓1.67%, NOx ↓ 27.01%, and CO↓19.15%
^35^	Engine operating parameters	LPG and diesel	Adaptive neuro fuzzy inference systems and ANN	Multi-objective optimization	Optimum conditions are determined as 194 bar injection pressure, LPG flowrate 1 LPM, and 1.13 BP, with overall R^2^ values of 0.99415
^36^	Fuel quantity calibration	Rapeseed methyl (B), Hydrogen (H) and water (W)	RSM	Multi-objective optimization	Optimum conditions are determined as B + 15H + 2.5W at 74.69% load. The corresponding performance indices were BSFC, BTE, NOx, HC, and CO emissions were 208.3 g/kWh, 39.2%, 941.2 ppm, 325.8 ppm, and 1073 ppm, respectively. Overall R^2^ values of 0.632, and the engine reaches the best state by fuelling
^37^	Engine parameters	Biodiesel blend	ANN and RSM	Desirability function approach	The optimum engine operating parameters are the biodiesel blend of 32% and 470 bar injection pressure R^2^ between 0.8663 and 0.9858. The relative error is less than 10%
^38^	Split injection parameters	Eucalyptus blend	RSM and DNN	Multi-objective optimization using the desirability approach	The optimized thermal efficiency was 33.7%, whereas BSFC is 0.37 kg/kWh. The reduced emission rates of HC, CO, NOx, and smoke are 6.74 ppm, 0.55%, 974 ppm, and 669 mg/m^3^ In contrast to the unoptimized condition, BTE ↑15.63% and BSFC↓5.12% with R^2^ values above 0.817

## Motivation and objective of present study

To achieve high levels of combustion efficiency in CI engines, a thorough examination of split injection behaviors of ammonia and biodiesel powered RCCI combustion is necessary. The literature indicates that the split injection map development of biofuels is being investigated. Furthermore, the performance and emission requirements of split injection calibration maps for ammonia and biodiesel dual fuel RCCI combustion have not yet been examined. To bridge this gap, the split injection behaviors of ammonia and biodiesel powered RCCI combustion on performance and emission characteristics were investigated. The knowledge gained could improve the way ammonia is utilized as a fuel for CI engines. Another novel component of this study was the application of statistical and numerical approaches (RSM and ANN) to develop optimal calibration model and predict the variables. Using these techniques reduces experimentation time and cost while improving system performance. RSM is statistics-based research that effectively creates relationships among the many input variables in order to produce the finest empirical model. In the present study, RSM was utilized to develop a regression model and choose the most effective optimal injection settings. In addition, an ANN model was developed to better predict the response under constant load conditions.

## Materials and methodology

### Methodology of intended work

The use of ammonia in CI engines requires optimal split injection calibration parameters with low emissions. Present study may help to adapt the electronic injector control settings across the full engine operation. First, a preliminary examination is performed on the chosen engine to determine the factors that have a major effect on the engine characteristics in the maximum and minimum limit variations. Following that, the whole calibration activity is interested in approaching the empirical model technique since the numerical method of investigation is used. It also enables the collection of a large amount of information on the influence of responses. The associated responses are documented after the experiment is completed in accord with the investigational plan. Data pre-processing is used to increase the model's prediction accuracy. The RSM then develops the polynomial model using the experimental observations. As a consequence, an optimal main injection map is created for a 2-step pilot mass ratio and a 1-step pre-injection angle using the multi-objective optimization technique. Furthermore, the ANN model is built using three subsets of accessible data (train, validate, and test). The optimal neuron weight and biases are calculated by an iterative technique with a continual trial-and-error procedure to predict the engine responses. The determination coefficient (R^2^) is used to assess the predictability of both models. Furthermore, high-efficiency optimal main injection map responses are used to generate the solution for all operational parametric combinations. The mathematical function-based optimisation desirability approach is then used to detect the desired engine responses. The estimated response is confirmed and scrutinised sequentially with respect to the intended optimisation function and applicable limitations. The optimisation point of main injection in this study is aimed at establishing an expected engine pilot mass and pre-injection angle. Finally, the split injection table is confirmed by inputting to the open ECU and comparing the amount of divergence from the projected optimal solution.

### Experimental test setup

A naturally aspirated water cooled four-stroke single-cylinder common rail engine was chosen for the experimental investigation. Table 3 describes the whole engine specification. The desired constant-speed, variable-load engine is properly installed, and the crank shaft is directly linked with a water-cooled eddy current dynamometer rated at 3.5 kW. The dyno control panel was manually adjusted to 80% of the maximum load capacity.

**Table 3:** Test engine specification.

Parameters	Details
Engine make	Kirloskar
No. of cylinder	1
No. of stroke	4
Cooling type	Water cooled
Stroke X Bore	110 × 87.5 mm
Displacement	661 cc
Rated power	3.5 KW@ 1500 rpm
CR	17.5
Dynamometer	Eddy current
Connecting rod length	234 mm

**Figure 1:**
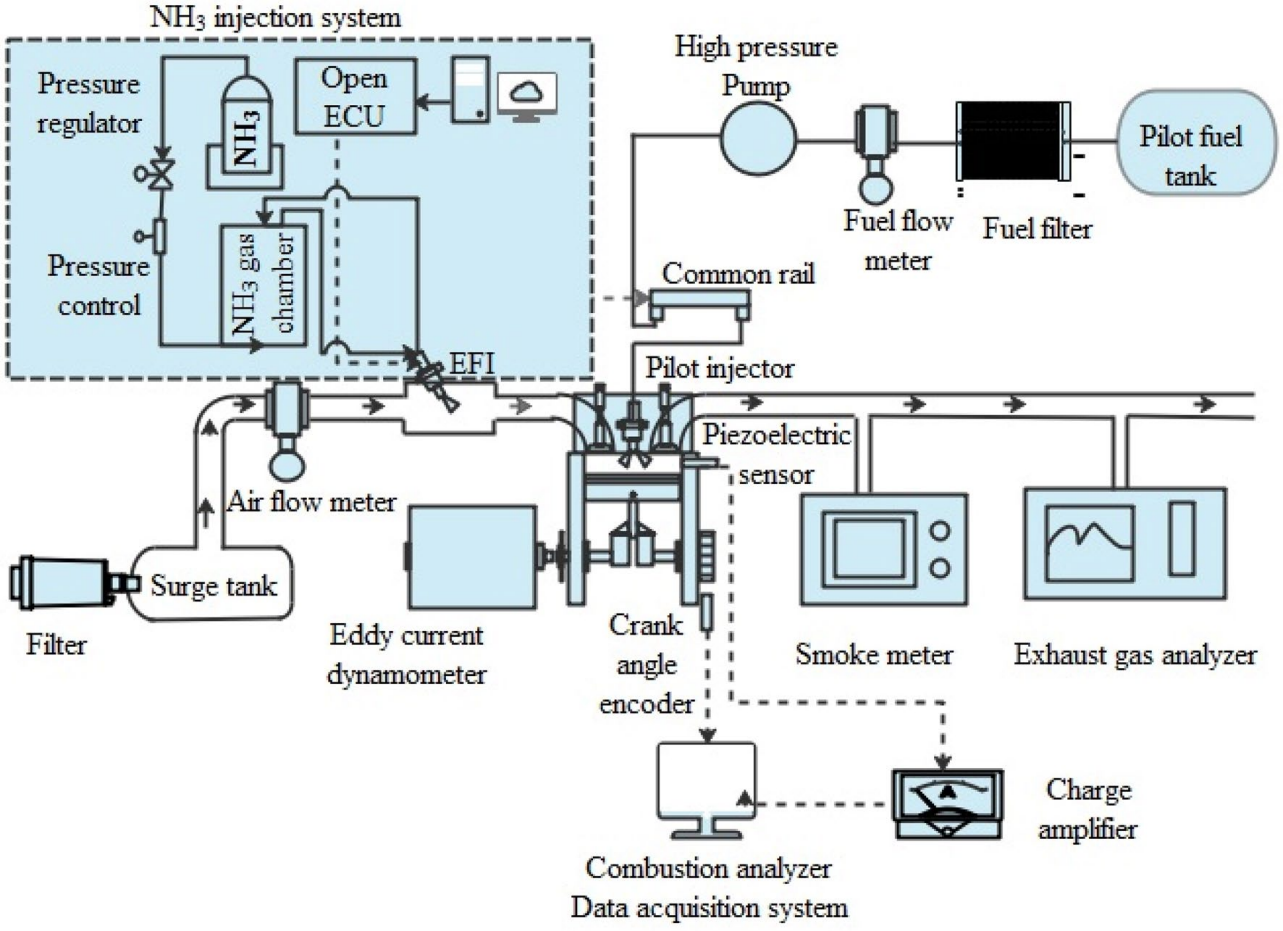
Experimental setup.

The noncontact type of speed sensor is used to detect engine RPM, while the strain gauge is employed to gauge the engine load. A bi-directional water-cooled eddy current dynamometer that is connected to the engine regulates the load. To keep the dynamometer and engine cylinder at the optimal temperature, a variable water-cooling arrangement is furnished with the circuit. Thermocouple sensors are correctly installed at the corresponding junctions to measure the engine temperature. Cylinder pressure at the crank angle position is required to study the combustion characteristics at each cycle. To get that data, the cylinder head is equipped with a piezoelectric sensor with a charge amplifier. Using an AVL crank angle encoder, cylinder pressure data from 100 stable consecutive cycles was collected for each operational point at a precision of 0.2 CAD. The calorimetric fuel flow and anemometer sensors record the engine's fuel consumption rates and air intake for performance studies. Figure 1 depicts a conceptual structure of the investigational engine setup. Furthermore, the engine control unit is changed to manual operational mode for experimentation, with a user defined capability for split injection timing parameters. To implement this capability, the NIRA launched the ECU, which is controlled by the MCAR program. To obtain information from all sensors, the NI Instrument USA acquisition unit of 250 kHz is employed. The engine software package makes use of the obtained data and computes the performance indices. Furthermore, thermodynamic rules are applied to determine different combustion and performance metrics. The engine-out emission concentration and the AVL-made DI gas analyzer measure the HC, CO, and NOx emissions concentrations, while the AVL-made smoke meter assesses smoke opacity. To prevent humidity and particles from exhaust gas, a moisture trap and filter setup are provided. The complete information associated with measurement and precision is given in Table 4. In addition, the uncertainty of all the equipment utilized in this inquiry for measuring different parameters is determined. The overall uncertainty of the investigation is within the acceptable limit of around 4.02%.

**Table 4:** Uncertainty.

Instrument	Sensor/principle	Range	Accuracy	% Uncertainty
Speed sensor	Inductive principle	0–5000 rpm	± 10 rpm	± 0.16
Load sensor	Strain gauge load cell	0–40 kg	± 1 g	± 0.18
Fuel flow	Calorimetric transmitter	0–10 bar dp	± 0.1 bar	± 0.3
Emission analyser	NDIR (non-dispersive infra-red)	CO: 0–10% vol	± 0.01% vol	± 2.14
	Flame ionization detector	HC: 0–10,000 ppm	± 10 ppm	± 1.12
	Chemiluminescence	NOx: 0–5000 ppm	± 10 ppm	± 2.13
Smoke meter	Blackening of filter paper	0–100%	± 1%	± 2.2

The aggregate uncertainty of the experiment is computed as follows:

$$\text{=}\sqrt{{0.16}^{2}\text{+}{0.18}^{2}\text{+}{0.3}^{2}\text{+}{2.14}^{2}\text{+}{1.12}^{2}+{2.13}^{2}\text{+}{2.2}^{2}\boldsymbol{ }}\text{=3.91}$$

### Investigational map development methodology

The critical components of calibrating a split injection system in an engine are proper identification of variables and responses. To design the factors and responses, the primary pertinent parameters influencing the optimum performance of the CRDi engine are examined based on the literature. Pre-injection timing, main injection timing and the pilot mass ratio are the independent engine control variables. The allowed range of each variable parameter is defined by previous observations of optimal engine functioning. Table 5 lists all of the chosen components and their ranges. The four-level versions in the input are used for numerical factors since there are more factors with higher levels, which allows for more permutations of variable levels. The design matrices are constructed using three numerical parameters. The top optimum design points are derived from a total of 64 DoE runs. The optimization of the split injection map entails the examination for the optimal trade-off result between the opposing goals of improved engine characteristics. The desired restrictions and goal operations of the three variable components and seven responses are defined using the desirability approach. In order to show a preference for lower or higher boundaries in respect to the objective, weight factors with values between 0.1 and 1 are utilized. In order to underline the degree of proportional contrast to the other replies, a 1–5 significance scale is also given. The details of the parameters utilized to search for the global optimal are listed in Table 6. Based on predetermined standards, the individualistic desirability repeatedly calculated for all engine variable combinations. The iteration with the highest "D" is often viewed as the reliable and most ideal combination of input variables that yields outcomes that are similar to or identical to those desired. The objective functions of response are estimated by Eqs. 1, 2, 3, and 4.

**Table 5:** Input factor levels.

Engine factors	Units	Type	Code	Levels		
				− 1	0	1
Pilot mass (PM)	%	Numeric	A	20	10	50
Main injection (MI)	Deg	Numeric	B	10	5	25
Pre-injection (PI)	Deg	Numeric	C	40	5	55

**Table 6:** Optimization criteria.

Name	Goal	Lower limit	Upper limit	Lower weight	Upper weight	Importance	Desirability
A: PM	Equal to 50	20	50	1	1	3	1
B: MI	Is in range	10	25	1	1	3	1
C: PI	Target to 55	40	55	1	1	3	1
BTE	Maximize	29.12	32.94	0.1	1	5	0.9056
BSEC	Minimize	13.129	14.39	1	0.1	4	0.786
HC	Minimize	151	185	1	0.1	3	0.7407
CO	Minimize	0.218	0.285	1	0.1	3	0.7402
NOx	Minimize	813	956	1	0.1	1	0.7506
Smoke	Minimize	34	42.5	1	0.1	3	0.7841
EGT	Minimize	277	348	1	0	3	0.8125
Combined	–	–	–	–	–	–	0.8027

1 $${d}_{i}\left({PI}_{i}\right)=\left\{\begin{array}{c}0if{PI}_{i}<{L}_{i}\\ \begin{array}{c}{\left(\frac{{PI}_{i}-{L}_{i}}{{T}_{i}-{L}_{i}}\right)}^{Wt}{ifL}_{i}\le {PI}_{i}\le {T}_{i}\\ {\left(\frac{{PI}_{i}-{U}_{i}}{{T}_{i}-{U}_{i}}\right)}^{Wt}{ifL}_{i}\le {PI}_{i}\le {T}_{i}\\ 0if{PI}_{i}>{U}_{i}\end{array}\end{array}\right.-\mathrm{Target function}$$

2 $${d}_{i}({BTE}_{i})=\left\{\begin{array}{c}0if{BTE}_{i}<{L}_{i}\\ \begin{array}{c}{\left(\frac{{BTE}_{i}-{L}_{i}}{{U}_{i}-{L}_{i}}\right)}^{Wt}{ifL}_{i}\le {BTE}_{i}\le {U}_{i}-\\ 0if{BTE}_{i}>{U}_{i}\end{array}\end{array}\right.\mathrm{Maximize function}$$

3 $${d}_{i}({Y}_{i}\left(BSEC, HC, CO, NOx, Smoke, EGT\right))=\left\{\begin{array}{c}0if{Y}_{i}(x)<{L}_{i}\\ \begin{array}{c}{\left(\frac{{Y}_{i}(x)-{L}_{i}}{{L}_{i}-{U}_{i}}\right)}^{Wt}{ifL}_{i}\le {Y}_{i}\left(x\right)\le {U}_{i}-\\ 0if{Y}_{i}(x)>{U}_{i}\end{array}\end{array}\right.\mathrm{Minimize function}$$

4 $$D={\left(\sum_{i=1}^{n}{d}_{i}{(Y}_{i}^{{r}_{i\dots n}})\right)}^{1/\sum {r}_{i}}-\mathrm{Overall Desirability}$$

## Engine assessment and the development of response models

**Figure 2:**
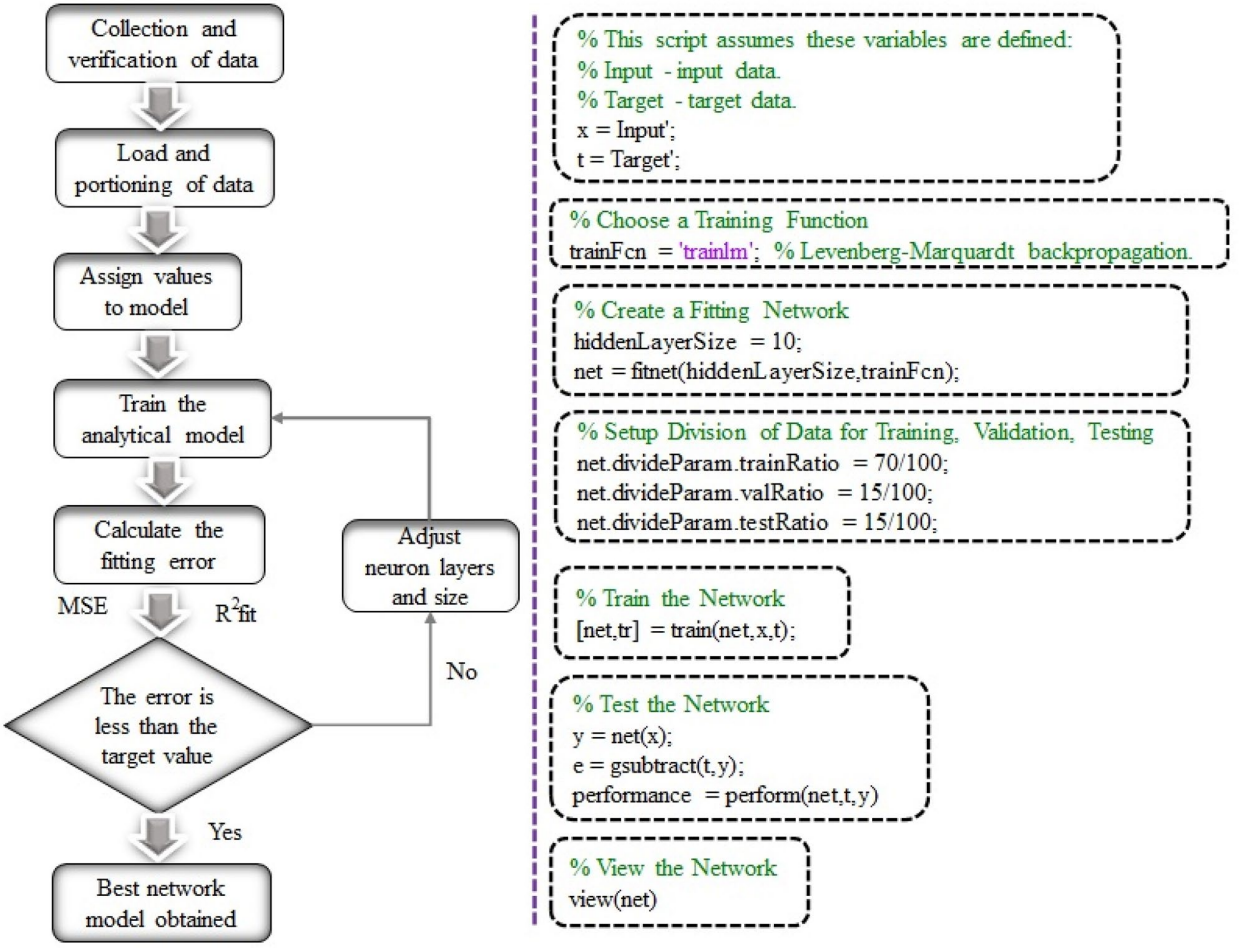
MATLAB operation sequence and algorithm order for the response prediction.

The responses are observed in line with the experimental design matrix. Individual regression models (RSM) and Global Neural Models (ANN) are constructed relying on the observed average engine responses. The ANN model was developed using the codes represented in Fig. 2. This section contains thorough information on model creation and model assessment. The following section provides complete information on map creation and model development assessment.

### Response surface method

The RSM process may be made more successful in such experimental research by decreasing both the cost and the time required. The RSM approach was used in this study using the Design Expert-12 software. RSM input factors and their levels are listed in Table 5. The design developed by RSM consists of 64 runs. After acquiring the investigational data with the RSM experimental plan, numerical modelling is implemented.

**Figure 3:**
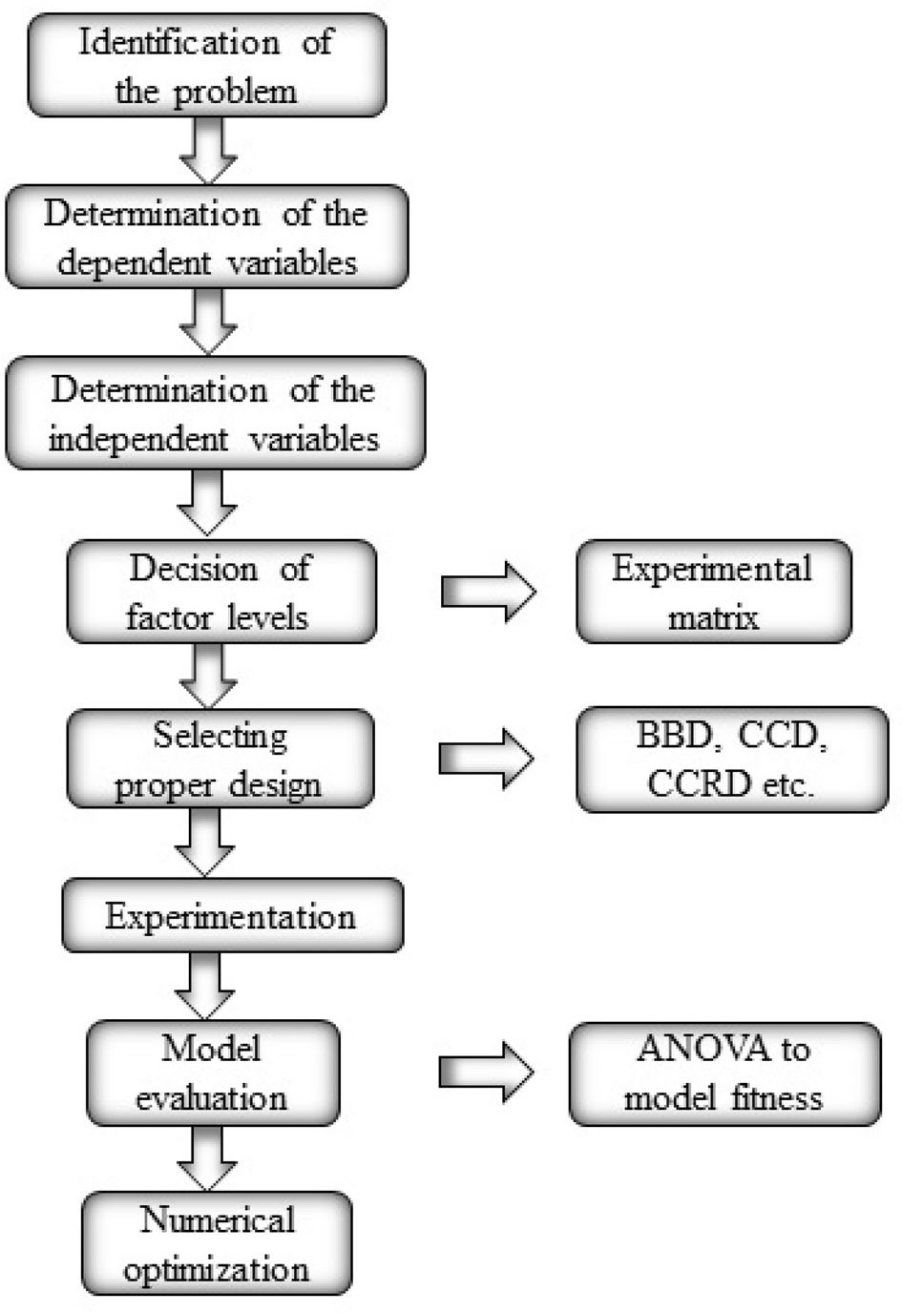
RSM sequence for engine response optimization.

Figure 3 shows the regression model development procedure. The last stage of the RSM approach is numerical optimization. During optimization, the intended conditions are inserted into the factors and responses, and the optimal independent factors and variables are identified.

The analysis of variance (ANOVA) is used to compare or correlate the effects of independent factors on a dependent variable for RSM regression modal. R^2^, Adj. R^2^, and Pred. R^2^ values were employed as correlation coefficients to evaluate the regression fit statistics. The additional diagnostic criteria for evaluating the developed response variable model are shown in Table 7. Both the reliability and adequacy of the model are shown by the correlation coefficient R^2^. The agreement of the model is shown by the difference between the adj. R^2^ and pred. R^2^ values. The acceptable precision (AP) number illustrates the model's accuracy. Table A (appendix) contains the established regression models for each response, where A stands for pilot mass, B for main injection angle, and C for pre-injection angle. As a result, the RSM analysis shows that split injection parameter in relation to the test circumstances was well anticipated.

**Table 7:** Model evaluation.

Parameters	BTE	BSEC	HC	CO	NOx	Smoke	EGT
Std. dev	0.0055012	0.0018057	0.1174934	0.0003003	0.3408255	0.028727	0.2259723
Mean	31.311452	13.710519	166.50313	0.2486491	889.19614	37.745363	308.32965
C.V. %	0.0175692	0.01317	0.0705653	0.1207565	0.0383296	0.0761074	0.0732892
R^2^	0.9999728	0.9999675	0.9998148	0.999676	0.9999203	0.9998317	0.9998392
Adjusted R^2^	0.9999611	0.9999534	0.9997349	0.9995362	0.9998859	0.999759	0.9997698
Predicted R^2^	0.9999354	0.9999184	0.9995411	0.9991804	0.9998025	0.9995819	0.9996001
Adeq precision	1235.367	1255.1665	529.33626	404.92208	745.20821	534.83739	561.40988

### ANN engine response model development

**Figure 4:**
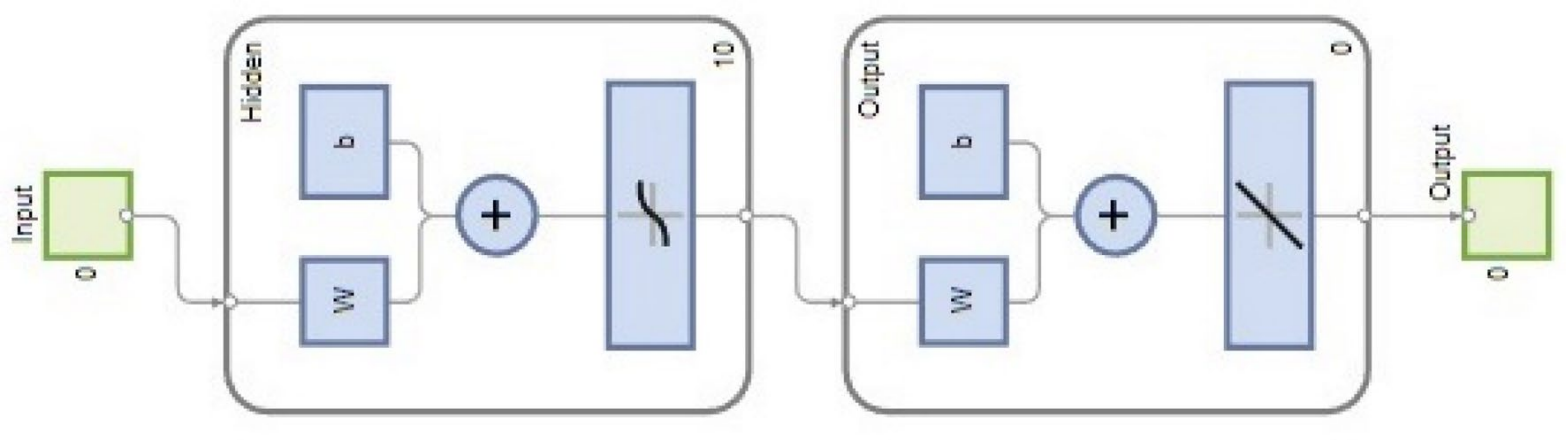
Schematic structure of ANN model.

A non-linear ANN approach is the most successful way to forecast the engineering system behavior. In this experimentation, MATLAB R2022a package software was used to perform the ANN model and the experimental data was utilized to create an ANN response model. In order to train the network, 70% of the data collected from the actual engine trials were utilized as input, while the remaining 30% of data were used to test and verify the ANN model. In this scenario, the network is composed of three layers: an output layer, a hidden layer, and an input layer. Using the trial-and-error method, the optimal number of neurons for the hidden layer was found. A total of 7 ANN models have been developed to anticipate the dependent variables of BTE, BSEC, HC, CO, NOx, smoke, and EGT. As illustrated in Fig. 4, the network consists of an input layer with three independent variables, a hidden layer with 15 neurons, and an output layer with one dependent variable. A multilayer, feed-forward, and back-propagation ANN model was utilized in this work. To achieve quick training and excellent accuracy, the Levenberg–Marquardt (TRAINLM) learning method was used. Based on the input variable, the TRANSIG algorithm aids in determining the responses.

The ANN model also carried out continuous training, testing, and validation for every response. The performance of the weights and bias settings for the network are selected using the MSE value. Using the coefficient of determination (R) shown in Table 8 as a consequence, the significance of the experimental results and the predicted results has been investigated. Because the acquired findings are so similar, the created ANN model could be capable of predicting the experimental results in real-time. Using source code, Fig. 2 shows the progression of the MATLAB approach from variables definition through neuron layer building, training, and assessment.

**Table 8:** Error analysis of ANN predicted data.

Response models	Training data (70%)	Test data (15%)	Validation data (15%)	Overall data (100%)
BTE	0.99998	0.99988	0.99998	0.99996
BSEC	0.99995	0.99988	0.99994	0.99994
HC	0.99996	0.99924	0.99995	0.99985
CO	0.99981	0.99946	0.99965	0.99972
NOx	1	1	1	1
Smoke	0.99995	0.99994	0.99995	0.99995
EGT	1	0.99999	0.99999	1

## Results and discussions

The study effort for the split injection map has resulted in improved engine output characteristics of ammonia premixing. The first portion of this section analyses the model's efficacy. Furthermore, the variation patterns of essential performances and emission characteristics pertaining to the ammonia optimal maps are addressed. The experimental validation findings are also presented to validate the optimization's reliability.

### Optimum split injection map from RSM

**Figure 5:**
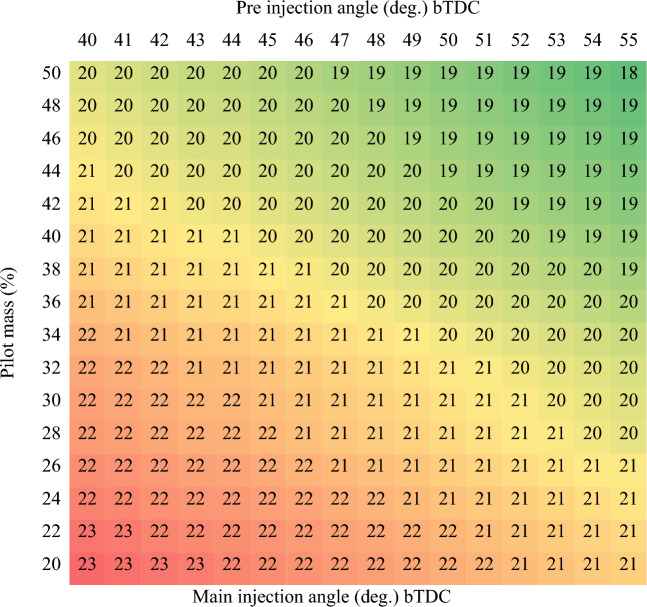
Split Injection control map using RSM multi-objective optimization approach.

The multi-objective optimization is connected with the Design Expert v12 program. The objectives of each response behavior are "maximization" of BTE and "minimization" of other responses. For the main injection angle, the "in range" goal is preferable. The "equal to" option is chosen as a pilot mass goal, while the "Target" option is for the pre injection angle at the fixed position. During numerical optimization, all aspects are given equal consideration. The optimal points are obtained by retaining the pilot mass at 50% (interval of two steps) and sweeping the pre injection from low to high (40 to 55) with a one-step interval. Similarly, the best solutions are extracted for each pilot mass requested. The optimal CRDi injection control for ammonia biodiesel fuel has been successfully identified based on the table shown in Fig. 5. When the pilot mass ratio increases, a retarded main injection angle provides greater thermal efficiency because the remaining fuel is injected later to manage an engine's power demand. Furthermore, pre-injected biodiesel reduces the ignition delay and automatically ignites when the cylinder reaches the auto-ignition temperature. Biodiesel acts as the ignition source; when the biodiesel ignites, ammonia starts to combust. The performance and emission phenomena as a consequence of ideal split injection settings as compared to unoptimized outcomes are reviewed in the sections that follow.

### Engine responses of optimal split injection map

#### Performance parameter

**Figure 6:**
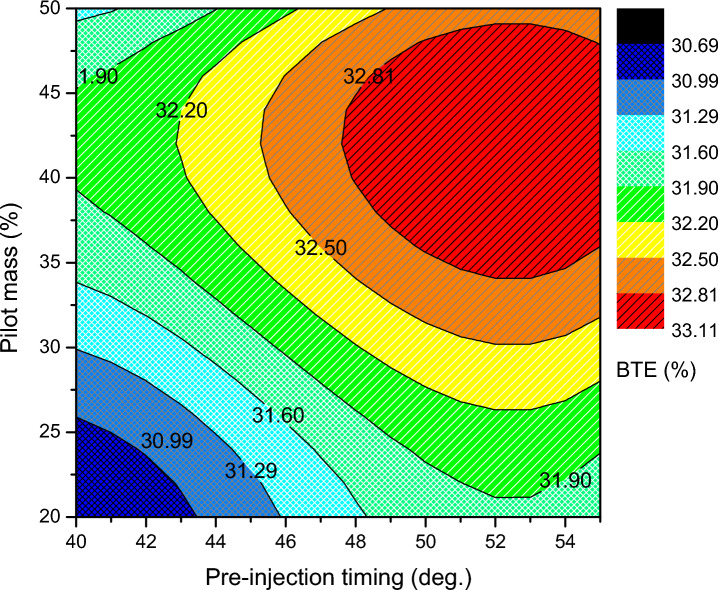
Brake thermal efficiency of split injection calibration map.

**Figure 7:**
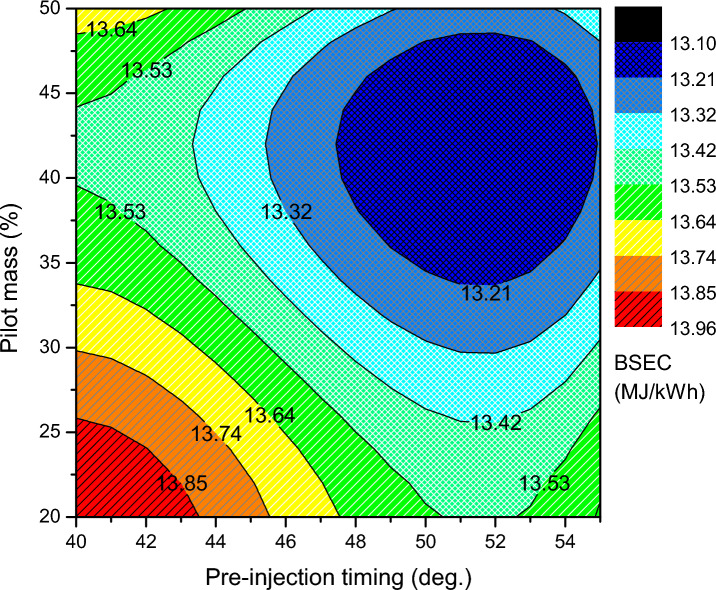
Brake specific energy consumption of split injection calibration map.

The BTE and BSEC of the optimal RSM map of ammonia-biodiesel-powered operation are shown in Figs. 6 and 7. BTE indicates how efficiently an engine converts chemical energy from fuel into mechanical power, and the BSEC trend was opposite the BTE trend. The region with the greatest BTE is between 38 and 44% pilot mass for pre-injection angles of 50 °CA to 54 °CA bTDC. Above that point, the BTE drops and the BSEC increases owing to the combined influence of pilot mass, per-injection, and main injection effect. In the calibration band zone, sufficient in-cylinder temperature produces effective combustion. At the same time, lower fuel consumption to provide more power leads to greater BTE. However, only the marginal drop in BTE occurs in the calibration map, which reveals well calibrated injector control settings. The highest BTE among the optimized values is 33.11%, which is the contribution of 42% pilot mass at 54 °CA bTDC of pre-injection and 19 °CA bTDC of main injection. At that point, the BSEC is reduced to 13.15 MJ/kWh. At lower pilot mass premixing, significant amount of fuel to be delivered at the main injection, hence, a shorter duration is accessible for the combustion, which reduces the period of vaporizing and air fuel mixing and thus reduces thermal efficiency. Further advancement of pre and main injection is not so advantageous; it leads to a fall in BTE. Pre injection is advanced farther, which has a detrimental compression effect while also affecting power output. The split injection control variables are calibrated taking into account all of these trade-off consequences without significantly penalized specific responses. As main injection further advances, due to the lower cylinder temperature, the charge takes longer to ignite and becomes more homogenous, which causes the diffusion combustion phase to lengthen and the thermal efficiency to decrease. As the advanced pre-injection timing, greater PRR at the end of the compression stroke raises the negative compression work. The thermal efficiency decreased due to a decrease in net work done and an increase in fuel consumption. Advancement of the injection time resulted in a rise in BTE and a commensurate reduction in energy consumption up to a point after which the trend changed. They claimed that the ideal split injection parameter for an engine to operate at its most efficient depended on the fuel's ability to burn.

#### Emission parameter

The region with the lowest emission is between 38 and 44% pilot mass for pre-injection angles of 50 °CA to 54 °CA bTDC. The emission parameters of HC, CO, smoke, and EGT were lowered to 151 ppm, 0.212% vol., 34%, and 278 °C, while NOx was increased to 955 ppm, which is the contribution of 42% pilot mass at 54 °CA bTDC of pre-injection and 19 °CA bTDC of main injection. Further advancement of pre and main injection is not so advantageous; it leads to a raise in emission. As main injection further advances, due to the reduced global temperature, the diffusion combustion phase increases, which increases the emissions. At advanced pre-injection timing, greater PRR at the end of the compression stroke raises the negative compression work leads to higher fuel consumption to generate same power leads to higher emissions. Advancement of the pre-injection timing caused a reduction in emissions up to a point after which the trend changed.

**Figure 8:**
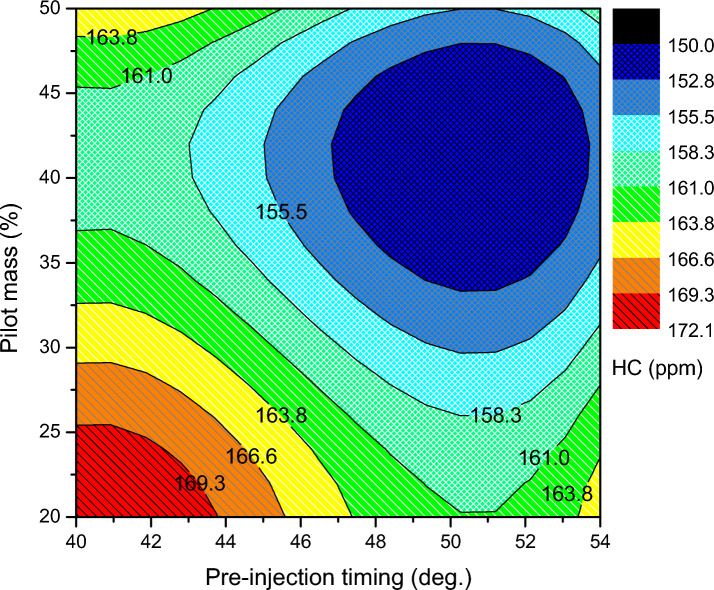
HC emission of split injection calibration map.

Hydrocarbons, or more accurately organic emissions, are a result of the hydrocarbon fuel's incomplete combustion. A greater quantity of HC emission is released for decreased pilot mass premixing. In the region of lower pilot mass premixing zone, providing more fuel in the main injection leads to a non-homogeneous mixture, which produces a leaner and richer combustion. Consequently, with unoptimized split injection parameters of pilot mass ratio 20%, pre injection at 40 °CA bTDC, and main injection at 10 °CA bTDC, the rich mixture in the local spots is not completely burned; in this zone, HC is emitted at an elevated level of 185 ppm. As the pilot mass increases, the exhaust hydrocarbons are slightly reduced, due to the lean charge mixture and adequate in-cylinder temperature to burn the fuel. Consequently, the lowest of 150 ppm of HC is emitted at pilot mass ratio 42%, pre injection timing at 52 °CA bTDC and main injection timing at 19 °CA bTDC. From Fig. 8, it is known that the advancement in pre-injection angle corresponds to the continued decrease in HC. Similar effects account for this trend. Additionally, at all operation regimes, the biodiesel is a contributing factor to the HC emissions because improper biodiesel mixing causes the combustion to occur later in the process, producing more HC.

**Figure 9:**
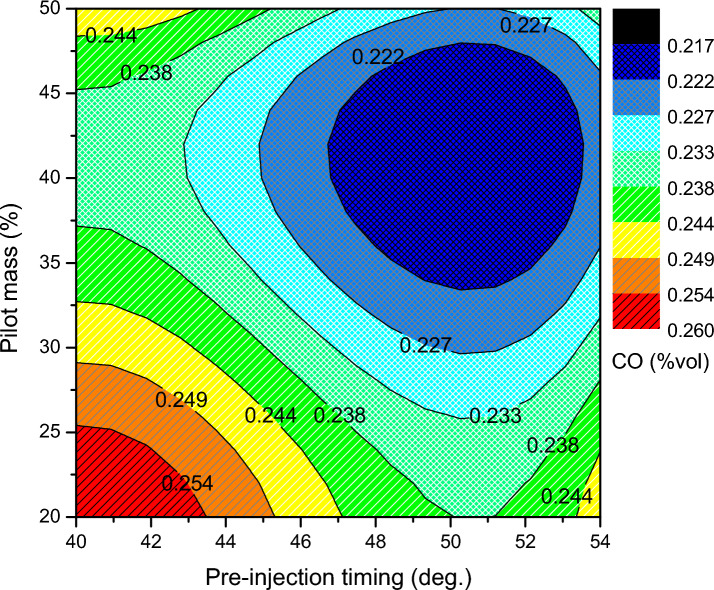
CO emission of split injection calibration map.

Carbon monoxide is produced in a combustion result when there is a shortage of oxygen to complete the combustion. Temperature and the availability of oxygen for combustion are key factors in the production of CO. The production of CO occurs when an engine is running at a low in-cylinder temperature and a fuel-rich equivalence ratio. A larger amount of main injection biodiesel causes the charge to be a rich mixture at a lower pilot mass premixing condition, which results in a higher quantity of CO being produced. Additionally, decreased in-cylinder temperature due to ammonia has a cooling effect that lowers the rate of fuel vaporization. A lesser quantity of CO is released in the event of higher pilot mass premixing. In that zone, a significant amount of fuel is injected prior to the cylinder's auto ignition temperature, which improves the ignition delay, and the thermal efficiency starts to increase. Figure 9 illustrates the minimum exhaust CO rate to be 0.285% volume, which is almost 21.4% less than the unoptimized split injection of 0.22% volume. This behavior is mainly caused by the combustible mixture's greater latent heat and decreased cetane properties. This trend is managed by varying the pilot mass premixing ratio and advancing pre-injection timing. The CO emission rates are strongly noticed during the whole engine operation for both circumstances, at lower pilot mass ratios and higher pilot mass ratios.

**Figure 10:**
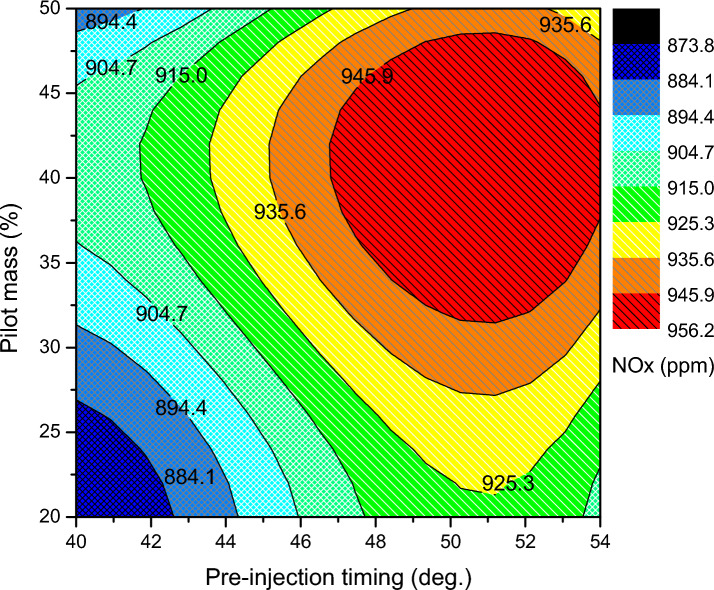
NOx emission of split injection calibration map.

Regarding the NOx emissions seen in Fig. 10, the optimized split injection parameter for pilot mass 44% at pre injection angle 53 °CA bTDC and main injection angle 19 °CA bTDC has a maximum of 956 ppm that is produced. But, for the unoptimized condition, lower rate of 813 ppm exists. Whereas the rate of NOx generation is significantly reduced with lower pilot mass ratios. The NOx tends to slightly rise when the pre injection angle is increased. This impact is mostly caused by the homogenous fuel mixture burning, which produces NOx through thermal action and ammonia bound N. NOx production decreases at lower pilot mass ratios due to incomplete fuel combustion. This is because premix-controlled combustion cannot occur because of the shorter ignition delay, which does not provide enough time for the fuel's vaporization and air fuel mixing. The detrimental effect arises at higher pilot mass ratios and advanced pre-injection timings because biodiesel has a lower latent heat of vaporization, a higher cetane number, and a higher diffusivity.

**Figure 11:**
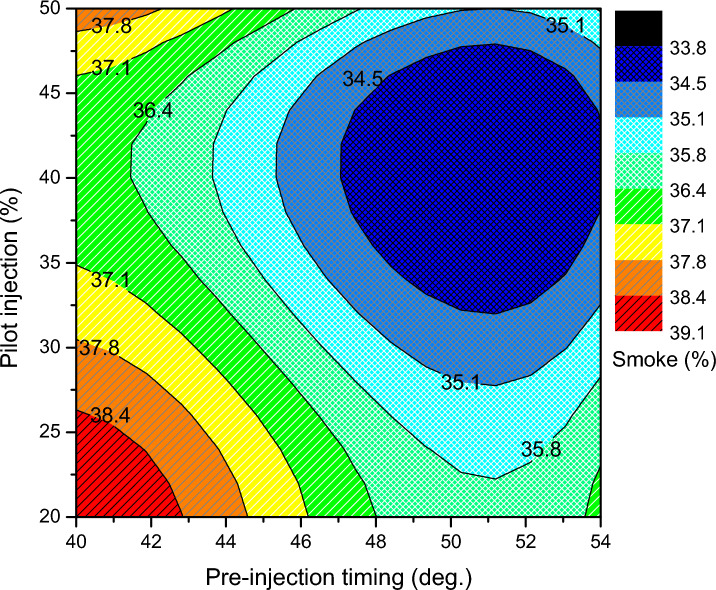
Smoke opacity of split injection calibration map.

By providing the correct amount of pilot mass with moderately advanced pre-injection, the optimization zone reduces smoke generation with respect to smoke concentration. A significant number of igniting centers are produced by the pilot mass's combustion in a premixed phase, expanding the reaction zone for the main injection. The concentration of smoke is greater in the unoptimized condition than it is in the optimized state. Substantial amounts of smoke are created because the unsaturated hydrocarbon with the larger carbon content is not oxidized at a lower pilot mass ratio combustion process. Subsequently, at retarded pre-injection, a greater fuel-to-air equivalency ratio causes the concentration of smoke to rise. Figure 11 demonstrates that, in comparison to all other regimes, smoke production is lower at higher pilot mass ratios and advanced pre-injection; this effect is primarily the result of the extended premixed combustion phase. But, in the region of greater pilot mass ratio and retarded pre-injection, there is more smoke. The higher volume of fuel at the main injection is associated with a drop in oxygen and fuel interaction. Moreover, there is an incredibly short duration accessible to combust the high-surged quantity. The emission pattern indicates that the adjustment of split injection parameters equalizes the normal NOx and smoke tradeoff impact. However, the NOx and smoke impacts of optimization are controllable to a certain degree, and the opposite effect is primarily dominating.

**Figure 12:**
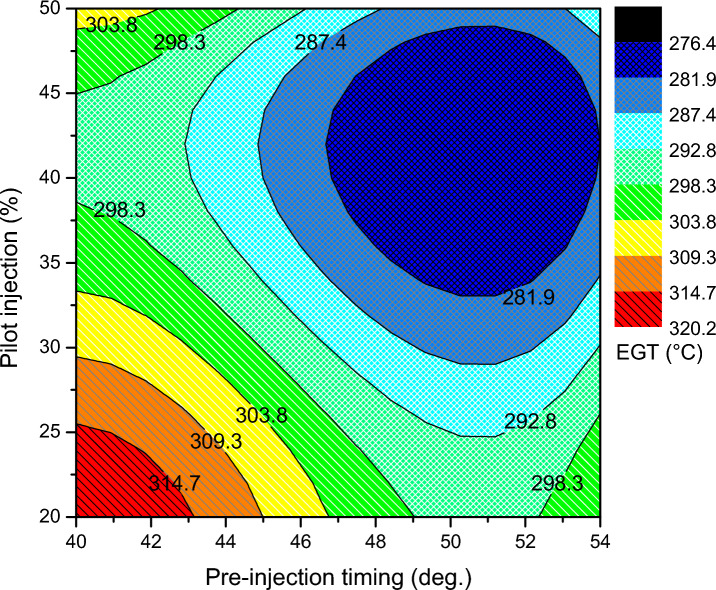
EGT of split injection calibration map.

Exhaust gas temperature shows how well a fuel uses its heat energy. The amount of heat energy from the fuel converted to work decreases due to heat loss in the exhaust system or an increase in exhaust gas temperature. In regard to EGT from Fig. 12, the optimized split injection parameter for pilot mass 42% at pre injection angle 52 and main injection angle 19 has a minimum of 276 °C that is produced. But, for the unoptimized condition, higher rate of 348 °C exists. For the lower pilot mass ratio and retarded pre injection timing, the spray penetration for biodiesel may be long for the higher quantity biodiesel at main injection, because the density and kinematic viscosity of biodiesel is higher, and this causes a poor atomization rate. Consequently, these factors increased the exhaust gas temperature. These may result from burning more fuel at greater loads to fulfill the need for power as well as the biodiesel's increased oxygen content, which enhances combustion at the diffusion stage and therefore may raise the temperature of the exhaust gas. The prolonged burn duration brought on by the greater amount of biodiesel injected during the main injection may also have contributed to this rise. Injected biodiesel fuel particles may not have enough time to burn entirely as a result of the delayed combustion. As a result, certain fuel combinations tend to burn during the latter stages of expansion, which leads to afterburning. An easy scale to use for determining the degree of afterburning is the exhaust gas temperature. And it was found that the EGT was noticeably greater than it was during the optimal split injection operation^40–42^.

### Experimental verification

To confirm the optimized split injection parameter, an engine experiment is performed under certain operating circumstances. For each operating point, three measurements are taken. The test mean value is validated with the findings and evaluates the improvements in engine characteristics, which are shown in Table 9. Furthermore, 42% pilot mass, 19 °CA main injection angle and 54 °CA pre-injection angle was selected for experimental validation, and optimized test values and experimental values were used for the comparison process. A properly adjusted map achieves a higher BTE while consuming lower fuel. The exhaust emissions are greatly decreased to demonstrate the capabilities of the constructed response map. Furthermore, the comparative error between the test and optimized values clarifies the regression model's dependability. It securely determines the ideal location with a relative inaccuracy of less than 5%. Equal weight constraints are applied to all replies since the relative errors, on the other hand, are within acceptable limits.

**Table 9:** Experimental validation.

Results	BTE	BSEC	HC	CO	NOx	Smoke	EGT
	%	g/kWh	ppm	% vol	ppm	%	°C
Pre-injection: 40 °CA bTDC, Main injection: 10 °CA bTDC, Pilot mass: 20%							
Un-optimized test value	29.12	14.39	185	0.285	813	42.5	348
Pre-injection: 54 °CA bTDC, Main injection: 19°CA bTDC, Pilot mass: 42%							
RSM Optimized value	33.11	13.15	151	0.22	955	34	278
ANN predicted value	33.05	13.22	152	0.221	952	34.1	280
Optimized test value	32.71	13.44	156	0.224	940	34.5	288
Enhancement (%)	12.33	6.60	15.68	21.40	15.62	18.82	17.24
RSM relative error %	1.21	2.21	3.31	1.82	1.57	1.47	3.60
ANN relative error %	1.03	1.66	2.63	1.36	1.26	1.17	2.86

## Conclusion

The intent of the present research is to increase the performance and reduce the emissions of a CI engine powered by ammonia and biodiesel to be on par with or better than conventional diesel fuel. The RSM multi-objective optimization technique is used to optimize the responses, and the ANN model is used to calibrate the split injection parameters. Based on the findings, the interpreted reasons for this study effort are as follows:

- The ANN approach produces a non-parametric empirical model with good prediction performance. Furthermore, it predicted various responses more accurately than the RSM parametric empirical model.
- The best injector control map was effectively framed by the RSM and self-built ANN prediction MATLAB code. This makes it easier to get an acceptable overall performance of alternate fuel in a CI engine.
- In overall comparison to the unoptimized map, the split injection optimized calibration map increases BTE by 12.33% and decreases BSEC by 6.60%, and the optimized map reduces HC, CO, smoke, and EGT emissions by 15.68%, 21.40%, 18.82, and 17.24%, while increasing NOx emissions by 15.62% under 80% engine load operation.
- RSM optimization with the most desirable level was selected for map development, and three trials were carried out to predict the calibrated map using ANN. According to the findings, the ANN predicted all responses with R > 0.99, demonstrating the real-time reproducibility of engine data better than the RSM model.
- The experimental validation of the predicted variables in the optimised map for 80% load has an error range of 1.03–2.86%, which is acceptable.
- The maps developed by this method will be preliminary maps that are loaded onto transient test automobiles. The created steady-state optimal map minimizes the boundary sweep range in the transitory examination procedure, reducing testing time and expense. Additionally, make it easier to exactly identify the engine ideal optimum spot.

### Supplementary Information

Supplementary Table 1.

## Data Availability

The datasets used and/or analyzed during the current study are available from the corresponding author on reasonable request.
