# Supplementary material for: Development of an ammonia-biodiesel dual fuel combustion engine's injection strategy map using response surface optimization and artificial neural network prediction
Source: Sci Rep. 2024 Jan 4;14:543. doi: 10.1038/s41598-023-51023-1 (PMC10767007; doi:10.1038/s41598-023-51023-1)
Supplement: Supplementary file 1 — Supplementary Table 1. [file 41598_2023_51023_MOESM1_ESM.docx]

Appendix

*Table A: Engine behaviour regression equation*

| **Response** | **Regression equation** |
| --- | --- |
| BTE | +32.07 +1.01 A+1.24 B+0.9184 C+0.0111 AB+0.0082 AC+0.0133 BC-0.5779 A²-0.4991 B²-0.2938 C²+0.0002 ABC-0.0139 A²B-0.0102 A²C-0.0074 AB²-0.0044 AC²-0.0088 B²C-0.0071 BC²-0.5917 A³-0.5205 B³-0.3903 C³ |
| BSEC | +13.38 -0.3407 A-0.3295 B-0.3724 C+0.0015 AB+0.0018 AC+0.0018 BC+0.2195 A²+0.2110 B²+0.1628 C²-0.0000 ABC-0.0023 A²B-0.0028 A²C-0.0023 AB²-0.0017 AC²-0.0027 B²C-0.0017 BC²+0.2157 A³+0.2075 B³+0.2220 C³ |
| HC | +157.32 -9.49 A-9.17 B-10.17 C+0.0984 AB+0.0849 AC+0.1118 BC+6.91 A²+5.39 B²+4.22 C²-0.0024 ABC-0.1928 A²B-0.1662 A²C-0.1143 AB²-0.0894 AC²-0.1298 B²C-0.1177 BC²+6.67 A³+5.15 B³+6.95 C³ |
| CO | +0.2303 -0.0180 A-0.0176 B-0.0191 C+0.0002 AB+0.0002 AC+0.0003 BC+0.0131 A²+0.0109 B²+0.0091 C²-7.379E-06 ABC-0.0005 A²B-0.0004 A²C-0.0003 AB²-0.0003 AC²-0.0003 B²C-0.0003 BC²+0.0125 A³+0.0103 B³+0.0130 C³ |
| NOx | +924.66 +29.38 A+49.89 B+40.79 C+0.2698 AB+0.2128 AC+0.6118 BC-21.56 A²-24.03 B²-18.24 C²+0.0063 ABC-0.6367 A²B-0.5022 A²C-0.2469 AB²-0.1874 AC²-0.5598 B²C-0.5388 BC²-21.95 A³-25.21 B³-21.38 C³ |
| Smoke | +35.60 -1.89 A-2.36 B-2.56 C+0.0195 AB+0.0196 AC+0.0471 BC+1.53 A²+1.26 B²+1.06 C²-0.0007 ABC-0.0540 A²B-0.0545 A²C-0.0184 AB²-0.0156 AC²-0.0447 B²C-0.0373 BC²+1.49 A³+1.19 B³+1.40 C³ |
| EGT | +291.23 -17.39 A-19.32 B-19.60 C+0.2629 AB+0.2262 AC+0.3117 BC+11.42 A²+10.61 B²+8.76 C²-0.0078 ABC-0.3913 A²B-0.3368 A²C-0.2639 AB²-0.2179 AC²-0.3129 B²C-0.3002 BC²+10.96 A³+10.03 B³+11.93 C³ |
